# Modulation of Key Physio-Biochemical and Ultrastructural Attributes after Synergistic Application of Zinc and Silicon on Rice under Cadmium Stress

**DOI:** 10.3390/plants10010087

**Published:** 2021-01-04

**Authors:** James Mutemachani Mapodzeke, Muhammad Faheem Adil, Dongming Wei, Heren Issaka Joan, Younan Ouyang, Imran Haider Shamsi

**Affiliations:** 1 Key Laboratory of Crop Germplasm Resource, Department of Agronomy, College of Agriculture and Biotechnology, Zhejiang University, Hangzhou 310058, China; jmapodzeke@gmail.com (J.M.M.); 11516093@zju.edu.cn (M.F.A.); 22016025@zju.edu.cn (D.W.); 21816229@zju.edu.cn (H.I.J.); 2 China National Rice Research Institute (CNRRI), Fuyang 311400, China; ouyangyounan@caas.cn

**Keywords:** rice, cadmium toxicity, zinc, silicon, ultrastructure, malondialdehyde content, alleviation

## Abstract

Excessive industrialization and the usage of pesticides plague the farming soils with heavy metals, reducing the quality of arable land. Assessing phytoavailability of cadmium (Cd) from growth medium to plant system is crucial and necessitates precise and timely monitoring of Cd to ensure food safety. Zinc (Zn) and silicon (Si) have singularly demonstrated the potential to ameliorate Cd toxicity and are important for agricultural production, human health, and environment in general. However, Zn-Si interaction on Cd toxicity alleviation, their effects and underlying mechanisms are still fragmentarily understood. Seven treatments were devised besides control to evaluate the single and combined effects of Zn and Si on the physio-biochemical attributes and ultrastructural fingerprints of Cd-treated rice genotypes, i.e., Cd tolerant “Xiushui-110” and Cd sensitive “HIPJ-1”. Supplementation of both Zn and Si promoted plant biomass, photosynthetic parameters, ionic balance, and improved chloroplast ultrastructure with minimized Cd uptake and malondialdehyde (MDA) content due to the activation of antioxidant enzymes in Cd stressed plants. The combined effects of 10 μM Zn and 15 μM Si on 15 μM Cd displayed a greater reduction in Cd uptake and root-leaf MDA content, while enhancing photosynthetic activity, superoxide dismutase (SOD) activity and root-leaf ultrastructure particularly in HIPJ-1, whilst Xiushui-110 had an overall higher leaf catalase (CAT) activity and a higher root length and shoot height was observed in both genotypes compared to the Cd 15 µM treatment. Alone and combined Zn and Si alleviation treatments reduced Cd translocation from the root to the stem for HIPJ-1 but not for Xiushui-110. Our results confer that Zn and Si singularly and in combination are highly effective in reducing tissue Cd content in both genotypes, the mechanism behind which could be the dilution effect of Cd due to improved biomass and competitive nature of Zn and Si, culminating in Cd toxicity alleviation. This study could open new avenues for characterizing interactive effects of simultaneously augmented nutrients in crops and provide a bench mark for crop scientists and farmers to improve Cd tolerance in rice.

## 1. Introduction

Cadmium (Cd), being a noxious inorganic contaminant with no involvement in any essential biological function, is a major yield-limiting environmental pollutant potentially damaging rice crop productivity [1,2,3]. In generality of heavy metal elements, Cd is rather more movable and toxic to organisms and is taken up readily by roots and translocated to shoots, thereby disrupting a wide range of cellular structures and plant metabolic processes essential for plant growth which is a cause of great concern [4,5,6]. Consequently, Cd transference from polluted soils into crops is inevitable and studies to reduce Cd toxicity in plants as well as lowering the accumulation frequency in edible plant parts are of immense importance to safeguard human health. Several environmental practices such as heavy industrialization and pesticides usage plague the farming soils with heavy metals reducing the quality of arable land. Additionally, previous reports have indicated that approximately 16% of Chinese agricultural lands were contaminated by heavy metals out of which more than 1.3 × 10^5^ ha^−1^ was adulterated with Cd [4]. Cadmium enhances lipid peroxidation which instigates toxicity by oxidizing nucleic acids, proteins and lipids leading to enzyme inactivation, chlorophyll concentration decline, decrease in photosynthetic rate, membrane damage, and in severe cases, cell death [6,7]. Rice is frequently consumed in Asia but compared to other cereal crops it has a propensity to accumulate more Cd and acts as a source of its exposure to the non-smoking population [8,9]. Strategies to alleviate Cd toxicity in plants include selecting genotypes that can effectively suppress root-shoot uptake of Cd and application of essential nutrients such as zinc, sulfur, and nitrogen fertilizers [10].

Zinc (Zn) regulates a wide range of biochemical processes and its homeostasis in plants is very crucial for promoting cellular development, thereby increasing plant biomass and yield [11,12]. Cd^2+^ and Zn^2+^ are found in the same group of the periodic table and share physico-chemical characteristics [10,13]; hence, synergistic or antagonistic effects can arise during ion uptake and translocation. The antagonistic effects between Zn and Cd could stem from shared transporters in the rhizosphere and xylem loading, creating a competition [14,15]. Recently, much attention has been drawn towards the nutrient management as a way to attenuate heavy metal toxicity. Most studies showed that Zn augmentation in soil reduced Cd accumulation in plants; for instance, application of Zn inhibited Cd absorption and translocation in barley under Cd stress [10,16]. It has been shown to be involved in alleviating Cd toxicity by increasing cell pigment contents and photosystem II efficiency in Cd exposed duckweed (*Lemna minor* L.) [17]. Zn addition is also attributed to improved rice growth and reduced oxidative stress against Cd instigated toxicity [18].

Second to oxygen (O_2_) (47%), silicon (Si) is the most abundant element on the Earth’s crust (27.7%), although in an inert form which is not procurable by plants [19,20]. Approximately 210–224 million tons of silicon is removed annually from arable/cultivable soil in the world [21,22]. Silicon improves plant tolerance against different biotic and abiotic stresses [23,24,25]. Under drought and salinity stress, Si increases plant growth, photosynthetic pigment concentration and grain yield [26]. It has a role of physico-mechanical barrier [27], and the formation of silica cuticle on leaves maintains water balance, avoids vessels compression, and decreases water loss caused by transpiration due to the reduction of stomatal diameter [8,28]. Moreover, it has a biochemical role; it improves osmolytes and antioxidant enzymes levels [29]. Previously, a study conducted on Brassica showed that Si-mediated Cd tolerance was mainly achieved by boosting antioxidant enzyme defense system and suppressing Cd uptake and root-to-shoot translocation [6]. Likewise, Si supplementation alleviated Cd toxicity by its immobilization, detoxification and reduced phyto-availability in growth medium due to an increased pH in maize [30]. Attenuation of Cd toxicity by Si has also been demonstrated in strawberry and *Echium amoenum* herb [24]. Furthermore, Si has been found to inhibit Cd accumulation in rice plants grown under 10 µM Si + 50 µM Cd, resulting in lower reactive oxygen species (ROS) and malondialdehyde (MDA) contents due to an enhanced antioxidant capacity [31]. Of note, the above-mentioned study also demonstrated a severe decrease in root hair length, altered leaf mesophyll cells, stomatal frequency, and vascular bundles under alone Cd stress, while Si supplementation mitigated these kinds of irregularities and sustained normal plant growth. Additionally, numerous reports have indicated the direct and indirect beneficial effects of Si against heavy metals related to plant growth and development [6,32], the co-existence of Zn and Si in medium also has evident benefits opposed to Cd toxicity in plants.

It is imperative to improve crop quality and food safety by maintaining the ionic balance of trace elements in crops and reduce the accumulation of toxic heavy metals. In our previous study [33], the long-term effects of Zn-Si on root to shoot mineral element translocation in mature rice plants under Cd stress were investigated and the analysis showed a significant reduction in Cd uptake, while the movement of elements such as Ca, Mn, and Mg varied in a genotype dependent manner. The objectives of current study were based on the hypothesis that combined Zn and Si in rice can effectively reduce Cd toxicity and improve rice growth from an early stage of plant growth. The hydroponics experiments were carried out with great emphasis on physiological characteristics, element dynamics, ultrastructural, and biochemical attributes which could be useful in providing a more convenient alternative for reducing Cd uptake in rice at organelle level.

## 2. Results

### 2.1. Plant Growth Attributes

**Figure 1:**
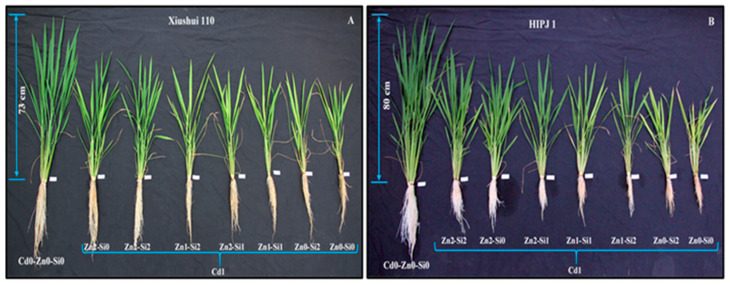
Differences in growth between two rice genotypes, Xiushui 110 (**A**) and HIPJ 1 (**B**) after 30-d treatment. Cd0 (0 μM), Cd1 (15 μM); Zn0 (0 μM), Zn1 (1 μM), Zn2 (10 μM); Si0 (0 μM), Si1 (5 μM), Si2 (15 μM).

The combined effects of Cd, Zn, and Si on the measured growth parameters are shown in Table 1 and Supplementary Table S1. There is an obvious genotypic as well as phenotypic variance between Xiushui-110 and HIPJ-1 in response to treatments (Figure 1). Relative to control, root length, shoot height, plant tillers, and dry weight of root, stem and leaf of all Cd treated plants were lowered in both genotypes regardless of Zn or Si supplementation. Addition of Zn managed to significantly alleviate the reduction in root length and shoot height of both genotypes. Si activity with Zn in combined treatments was more effective in HIPJ-1. The Zn-Si effect in Cd1-Zn2-Si2 treatment for HIPJ-1 performed overall better in all growth parameters as compared to other treatments under Cd stress, while Xiushui-110 did not have the same effect. The average production of tillers per plant stayed close to control plants in Xiushui-110 under Cd1-Zn2-Si0 and a same pattern was observed for the root dry biomass as well, contrary to HIPJ-1.

### 2.2. Photosynthetic Parameters and Relative Chlorophyll Contents

**Figure 2:**
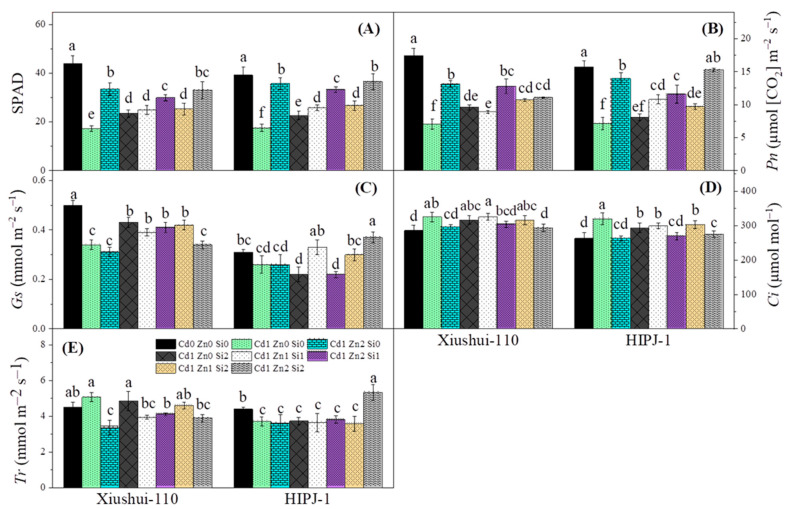
Effects of individual Cd, Zn, and Si treatments and their combination on (**A**); SPAD (soil and plant analyzer development), (**B**); net photosynthetic rate (*Pn*), (**C**); stomatal conductance (Gs), (**D**); intercellular CO_2_ concentration (*Ci*) and (**E**); transpiration rate (*Tr*) in two rice genotypes. Legends: Cd0 Zn0 Si0 (CK). Cd0 (0 μM), Cd1 (15 μM); Zn0 (0 μM), Zn1 (1 μM), Zn2 (10 μM); Si0 (0 μM), Si1 (5 μM), Si2 (15 μM). Vertical bars represent the means of three independent replicates (±SE). Different letters indicate statistically significant differences at *p* ≤ 0.05 probability level within the genotype.2.3. Antioxidant Enzyme Assay and Lipid Peroxidation.

The effects of Cd, Zn, and Si was assessed on relative chlorophyll content (expressed as SPAD value) and subsequent photosynthetic parameters illustrated in Figure 2. Cd increased plant stress and significantly inhibited plant growth by reducing photosynthetic rate (*Pn*) and relative chlorophyll contents (Figure 2A,B). Zn and Si addition independently, and in combination, significantly alleviated Cd stress and increased SPAD and *Pn*. However, both genotypes showed lower SPAD and *Pn* in treatment Cd1-Zn2-Si1 than Cd1-Zn2-Si0, which requires further exploration. Only HIPJ-1 restored to normal *Pn* under alleviation of combined Zn2 and Si2. In Xiushui-110, *Gs* was significantly reduced by the addition of Cd and Zn relative to its control but addition of Si caused a significant improvement in this regard. For HIPJ-1, *Gs* was significantly increased in Cd1-Zn2-Si2 followed by Cd1-Zn1-Si1 treatment (Figure 2C). Interestingly, Xiushui-110 showed overall higher *Gs* in treatments than HIPJ-1 indicating clear difference between the two genotypes on stomatal control stability and Xiushui-110 being a more tolerant genotype. Retention of *Ci* was significantly higher in Cd alone treated plants under Cd1-Zn0-Si0 for both genotypes and under Cd1-Zn1-Si1 treatment, particularly in Xiushui-110. From results, it is obvious that Zn and Si addition reduced intercellular CO_2_ concentration with treatments Cd1-Zn2-Si0 and Cd1-Zn2-Si2 closest to the control (Figure 2D). In HIPJ-1, the plants under Cd1-Zn2-Si2 treatment showed a higher transpiration rate (*Tr*) compared to its relative control, while Xiushui-110 exhibited *Tr* values under Cd1-Zn0-Si2 and Cd1-Zn1-Si2, closer to control indication a dose dependent response (Figure 2E).

**Figure 3:**
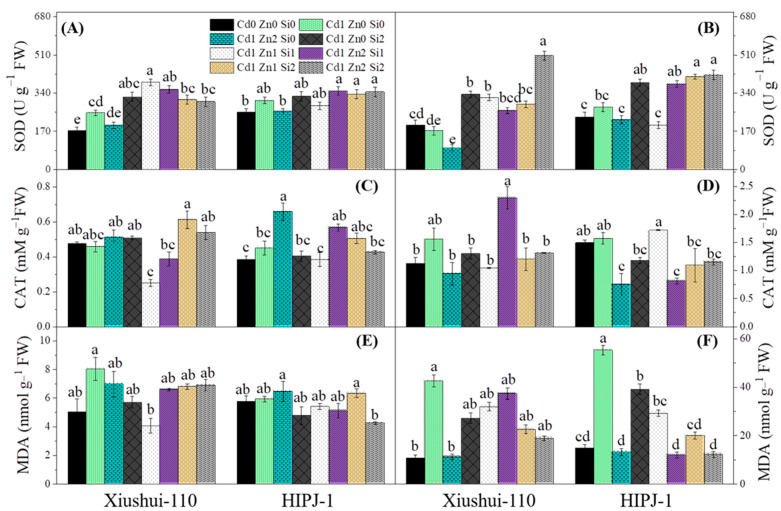
Effect of alone and combined stress of Cd, Zn, and Si application on antioxidant enzyme activities of superoxide dismutase (SOD) ((**A**): Root, (**B**): Shoot), catalase (CAT) ((**C**): Root, (**D**): Shoot) and malondialdehyde (MDA) contents ((**E**): Root, (**F**): Shoot) of two rice genotypes. Legends: Cd0 Zn0 Si0 (CK). Cd0 (0 μM), Cd1 (15 μM); Zn0 (0 μM), Zn1 (1 μM), Zn2 (10 μM); Si0 (0 μM), Si1 (5 μM), Si2 (15 μM). Vertical bars represent the means of three independent replicates (±SE). Different letters indicate statistically significant differences at *p* ≤ 0.05 probability level within the genotype.

Figure 3 shows root and leaf enzyme activities and MDA contents. Cd addition significantly increased root superoxide dismutase (SOD) activity in both genotypes (Figure 3A). Addition of Zn restored root SOD activity under Cd stress towards the control and significantly lowered leaf SOD. On the contrary, Si addition in treatments caused an increase in both root and leaf SOD (Figure 3B). For both genotypes, combined Zn and Si treatments had ultimate increases in leaf SOD as compared to Cd1-Zn2-Si0 treatment. Zn alone application caused a significantly high increase in root catalase (CAT) activity but in combined Si treatments root CAT was lowered. The lowest root CAT activity for both genotypes under Cd stress occurred in treatment Cd1-Zn1-Si1 using Zn-Si effects. As expected, Cd stress increased leaf CAT. Contrary with root CAT activity, Zn and Si alleviation (Cd1-Zn2-Si0, Cd1-Zn0-Si2) significantly reduced leaf CAT activity for both genotypes when compared to Cd alone treatment (Figure 3C,D). As expected, Cd stress instigated an increase in lipid peroxidation due to high root and leaf MDA values for both genotypes compared to control. It was evident that the treatment Cd1-Zn2-Si2 in HIPJ-1 reduced both root and leaf MDA contents but for Xiushui-110 reduction in root MDA contents were not much pronounced. Zn and Si managed to lower leaf MDA in both genotypes as compared to Cd sole treatment. However, when comparing treatments Cd1-Zn2-Si0 and Cd1-Zn2-Si1, leaf MDA increased in Xiushui-110 even though the values were not statistically different indicating that low Si increased MDA in that genotype and not HIPJ-1 (Figure 3E,F).

### 2.3. Elemental Profile and Cd Translocation Factor

The internal concentrations of Cd, Zn, Si, Ca, Mg, K, and Fe in roots, leaves, and stem-sheath at 30-d after treatment are shown in Table 2 and Supplementary Table S2. As expected, Cd and Zn concentrations in roots greatly surpassed the stem and leaf concentrations by 148% and 50%, respectively, with the exception of Si which alternatively increased in shoot than root under Cd stress. The observed increase in Cd content in plant tissue followed the trend root > stem-sheath > leaf. Between the two genotypes, Xiushui-110 had higher Cd in tissue as compared to HIPJ-1. Addition of sole 15 μM Si significantly reduced Cd content in root, stem-sheath and leaf for Xiushui-110. For HIPJ-1, sole Si reduced Cd levels in stem-sheath and leaf slightly except for root. On the other hand, Zn addition increased Cd concentration in tissues for both genotypes and addition of 5 µM Si (Si1) with 10 µM Zn (Zn2) spearheaded Cd increase in root and stem-sheath but 15 μM Si addition counteracted these effects. However, the addition of zinc Zn1 and Zn2 proved more efficient in HIPJ-1 as seen by the lower root Cd content as compared to sole Si alleviation. Combined Zn and Si was effective at reducing Cd accumulation in leaves when compared to sole Zn alleviation for both genotypes with 15 μM Si being most influential. Observing the treatment Cd1-Zn2-Si2 as compared to Cd1-Zn2-Si0 for both genotypes show that in HIPJ-1, Cd1-Zn2-Si2 significantly lowered stem-sheath Cd content in response to Si but in Xiushui-110 it was the opposite.

The concentration of calcium was observed to be increased under Cd1 Zn0 Si2 in roots and stem-sheaths of Xiushui-110, in comparison to its control, while in its leaves showed highest Ca contents under Cd1 Zn2 Si1 treatment. Although, under most of the treatments there were no differences in Ca contents in leaves of this genotype, highest values were obtained from plants grown under Cd1 Zn2 Si1 and lowest under Cd1 Zn2 Si0. In case of HIPJ-1 roots, except for Cd alone and Cd1 Zn2 Si2 treatments, where significantly higher contents were found; no statistically significant difference was witnessed. This genotype had Ca concentrated mostly in its leaf-sheaths under Cd1 Zn0 Si2, while its leaves faced a drop in Ca concentration under Cd1 Zn1 Si1, Cd1 Zn2 Si1, and Cd1 Zn2 Si2. Magnesium forms the central part of the chlorophyll molecule and its concentration was increased under Cd1 Zn0 Si2 in leaves of both genotypes (Supplementary Table S2). The concentration tended to increase under all treatments in roots and leaf-sheaths of both genotypes when compared to their respective controls. Moreover, the K contents did not vary significantly in leaves of Xiushui-110, HIPJ-1 displayed a significant increase under all treatments, noticeably under Cd1 Zn0 Si2. Treatments where Zn and Si were applied together had a positive impact on retaining the K concentrations close to control in stem-sheaths of both genotypes and a pronounced increase in its contents were observed under all treatments, particularly under Cd1 Zn2 Si0, compared to their respective controls in root tissues. Although Fe is not used in the synthesis of chlorophyll, it is essential for its formation and the addition of Cd negatively impacts its concentration. Interestingly, under all treatment and specifically under Cd1 Zn2 Si1 in Xiushui-110, the Fe contents increased in roots (Supplementary Table S2), while a reverse trend was observed in roots of HIPJ-1. Both genotypes displayed a decline in Fe concentration in their leaf-sheaths and leaves (Supplementary Table S2).

The effects of Cd, Zn, and Si treatments on Cd translocation factor (root to stem, stem to leaf TF%) are presented in Table 3. Cd root to stem TF% and stem to leaf TF% was genotype dependent. For instance, Cd root to stem translocation was higher in HIPJ-1 than Xiushui-110 with exception of treatments Cd1-Zn0-Si2 and Cd1-Zn2-Si2 while the Cd stem to leaf translocation was overall higher in Xiushui-110 than in HIPJ-1. Si alone alleviation was able to lower Cd translocation factor from stem to leaf in both genotypes and from root to stem in HIPJ-1 only as compared to Cd alone treatment. In Xiushui-110, high Si (Si2) was able to lower Cd TF from stem to leaf when compared to subsequent treatments with Si1. The combination treatment of Si2 with Zn2 successfully lowered Cd root to stem TF in HIPJ-1 as compared to Zn2 sole alleviation. Furthermore, Cd stem to leaf TF was also significantly lowered by Si in HIPJ-1 but slightly increased in Si2 and Zn2 combination. However, Zn increased Cd TF in both genotypes.

### 2.4. Ultrastructural Fingerprints

**Figure 4:**
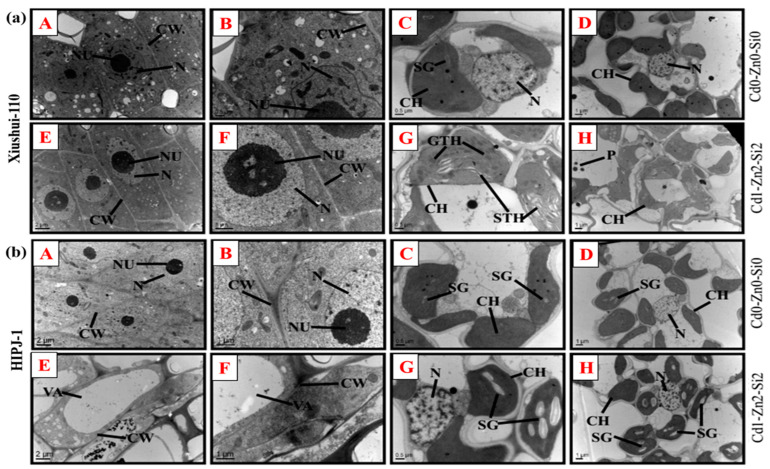
Ultrastructure micrograph images for (**a**) Xiushui-110 and (**b**) HIPJ-1 after 30-d treatments. Labels (**A**,**B**) (meristematic cells at root tip), (**C**,**D**) (leaf chloroplast) of control plants. Labels (**E**,**F**) (meristematic cells at root tip), (**G**,**H**) (leaf chloroplast) of treated plants Cd0 (0 μM), Zn0 (0 μM), Si0 (0 μM), Cd1 (15 μM), Zn2 (10 μM) and Si2 (15 μM). Legends. CH- chloroplast, STH- stromal thylakoids, GTH-granal thylakoids, SG- starch granule, P-plastoglobuli, CW-cell wall, N-nucleus, NU- Nucleolus, VA-vacuole.

The electron micrographs taken for the genotypes (a) Xiushui-110 and (b) HIPJ-1 are illustrated in Figure 4, showing meristematic cells at root tip (Figure 4A,B—control; E,F—Cd1-Zn2-Si2 treated), and leaf chloroplast (Figure 4C,D—control; G,H—Cd1-Zn2-Si2 treated). In root tips, Cd significantly affected root production by causing irregular root cell formation of which in Xiushui-110 the nucleolus was invisible in most cells and in HIPJ-1, which is Cd sensitive had all root tip meristematic cells destroyed as seen clearly in treatment Cd1-Zn0-Si0 compared to the corresponding control Cd0-Zn0-Si0 (Figure 4A,B and Supplementary Figure S1). Nevertheless, Si2 addition in Zn-Si combined treatments was able to retain root meristematic cells from the plasmolyzed state caused by Zn. Remarkably, formation of large vacuoles in root tip cells occurred highly in Cd1-Zn2-Si2 treatment for HIPJ-1 (Figure 4E,F). A clear substantial damage in chloroplasts occurred under Cd stress in both genotypes relative to the control. Disintegration of chloroplast membranes, reduced number of chloroplasts and plastoglobuli resulted in treatment Cd1-Zn0-Si0 for both genotypes when compared to control Cd0-Zn0-Si0 (Figure 4C,D and Supplementary Figure S1). Zn and Si markedly improved chloroplast formation, whereas the formation of plastoglobuli was higher with Si than Zn in both genotypes. Starch granule formation also increased significantly by Zn and Si addition. In Xiushui-110, Zn alleviation plainly caused fragmentation of stromal thylakoids (Figure 4G,H). Interestingly, cumulative Zn and Si combined in treatments made different chloroplast formations for Xiushui-110 with an increase in Si addition affecting the thylakoid system resulting in highly visible granal thylakoids even though plastoglobuli formation was reduced.

## 3. Discussion

Rice is an important crop in the world and due to its vulnerability towards preferential uptake of Cd, the toxicity caused by this heavy metal is quite detrimental to its growth and development. Accordingly, the current study explored the effects of Zn and Si on Cd toxicity in rice at vegetative stage. The addition of Cd reduced plant growth as shown with all measured growth parameters for both genotypes which support previous findings in barley [1,2,6], and wheat [34,35]. Zn addition managed to significantly alleviate the growth inhibition caused by Cd in both genotypes which also collaborate with previous studies in rice where Zn improved plant biomass and chlorophyll concentration [18,36], as well as the tillering and panicle formation [33]. Likewise, Si significantly had a positive impact on growth parameters which is in line with previous literature on wheat [37] and rice [31], but was strongly dependent upon genotype in combined treatments.

Interestingly, the combined Zn-Si (Cd1-Zn2-Si2) treatment mainly favored HIPJ-1 against Xiushui-110 in alleviating Cd stress which shows a different response mechanism (Figure 1). Si improves growth by blocking active apoplastic movement of Cd and its effects in plants [8,31,38], whereas Zn enhances Cd sequestration [14,15,36]. In contradiction with the results obtained from the long-term exposure of Cd, Zn, and Si treatments [33], the beneficial effects of Si on overall biomass were quite pronounced at vegetative stage of HIPJ-1. Furthermore, a previous cytological study on rice stated that Zn alleviated Cd in cooperation with Si by the formation of Si-hemicellulose matrix-Zn complexes that restrict Cd uptake [39]. Undeniably, to counteract Cd toxicity, the individual and combined treatments of Zn and Si greatly increased SPAD values (relative chlorophyll content) and *Pn* as compared to Cd alone treatment [40,41], which is in correspondence with our previous study [33], indicating the favorable impacts of Zn and Si on rice metabolism and stabilization of redox homeostasis in photosynthetic machinery. HIPJ-1 restored back to normal *Pn* with Cd1-Zn2-Si2 treatment while in Xiushui-110 the change was not pronounced. The increase in *Gs* upon the same treatment can justify our results as Si enhances stomatal control stability (*Gs*) which is in collaboration with work done by Liang et al. [42]. Previous study did not find significant difference in intercellular CO_2_ concentration (*Ci*) of rice under Cd stress [43], but the current study, on the contrary, showed that *Ci* increased under Cd alone treatment Cd1-Zn0-Si0 as compared to corresponding control Cd0-Zn0-Si0 (Figure 2). This increase could be a stimulated response caused by a higher retention of CO_2_ due to lowered photosynthetic rates caused by Cd. Additionally, the combined Zn-Si treatments reduced the internal CO_2_ concentration by utilizing CO_2_ at a higher rate to counteract the effects of Cd induced *Pn* deficits and improve plant stress tolerance in long-term studies as well [33]. The increased *Tr* upon Zn and Si interaction correlates with high photosynthesis and mineral element uptake (Supplementary Table S2).

Superoxide dismutase (SOD) and catalase (CAT) are very crucial metabolic enzymes for plants attributed to the maintenance of ROS balance [8], which justifies the results observed in the present study. The data reported here show Si instigating the increase of root and shoot SOD activity in both genotypes which support previous findings [6,33]. Da Cunha and Do Nascimento [38], demonstrated that Si has a stimulating mechanism for the elimination of free radical induced damage by enhancing antioxidant enzyme systems in Cd stressed plants. Conversely, Zn markedly lowered SOD activity in leaves and roots for both genotypes under Cd stress [36], but in combined treatments Si stimulated SOD production in roots and leaves, which is in line with our previous results [33]. CAT catalyzes the decomposition of H_2_O_2_ in tissues and Si has been found to reduce the H_2_O_2_ content in *Brassica chinensis* L. by increasing CAT activity [6]. However, in the present study, a decreased CAT activity in leaf occurred for both genotypes with Zn or Si alone application compared to their corresponding Cd alone treatments which could be attributed to corresponding lower H_2_O_2_ in leaves but in roots Zn alone increased CAT activity. Noticeably, Zn could enhance or attenuate CAT activity depending on genotypes, tolerance to heavy metals, plant tissues, and most likely the duration of treatment [33]. Moreover, Cd stress resulted in an increased MDA (an index for lipid peroxidation) content in the present study. Xiushui-110 had the highest root MDA content under Cd alone stress (Figure 3E), which could relate to the increased root Cd content (Table 2). In the Cd1-Zn2-Si2 treatment, a substantial reduction in root MDA and corresponding leaf MDA content for HIPJ-1 could relate to Si and Zn effectively inhibiting Cd uptake; the phenomenon which persists in plants till the maturation stage [33]. Thus, combined Zn-Si facilitated the maintenance of root tissue and subverted ROS damage by reducing lipid peroxidation and increasing antioxidant enzyme activities [33]. The treatment Cd1-Zn2-Si2 for HIPJ-1 utilized the ultimately increased SOD activity and greatly lowered MDA contents in both roots and shoot for growth improvement than all other treatments which prove the usefulness of Zn-Si combined effects in that genotype at vegetative stage.

Furthermore, in the current study, we noted the significant reduction of Cd upon Si supplementation as seen in previous literature [31,37,41]. Elevated Si levels proved to be useful for the reduction of stem-sheath and leaf Cd concentration in HIPJ-1 with the exception of roots, whereas in case of Xiushui-110 the reduction in Cd concentration occurred in all recorded plant parts, which supports the genotypic variation in their response to Cd. However, the addition of Zn1 and Zn2 proved more efficient in HIPJ-1 as seen by the lower root Cd content compared to sole Si application. The plausible reason being the addition of Zn having antagonistic effects against Cd, thereby competing for the same root uptake channels in rhizosphere [14,15]. Mehrabanjoubani et al. [44] also showed that supplies of 50 g L^−1^ Zn ultimately increased plant growth and higher tissue production in rice, which could be due to the genotypic difference or optimized plant metabolic responses to stress. Although, the combined Zn and Si treatments lowered Cd accumulation in leaves of both genotypes when compared to Zn alone, Si2 was found to be more influential. The reduced stem-sheath Cd content in HIPJ-1 by treatment Cd1-Zn2-Si2 as compared to Cd1-Zn2-Si0 must have caused the improved biomass (Table 1) in the same Zn-Si treatment with a contrasting response in Xiushui-110, providing an evidence depicting the genotypic difference. It is clear that the concentrations of Cd and Zn in roots exceeded the stem and leaf concentrations implying that plants have their own defense mechanisms to reduce heavy metal uptake and translocation in plant tissues. In addition, it is interesting to note that Si concentration was higher in shoot than root under Cd stress and similar trends have been reported in wheat [37], and rice [43,45].

Translocation factor is an essential way to track the uptake of heavy metals from root to shoot as it reflects the plants ability to increase or reduce metal transport [8], with high TF reflecting increased Cd movement in plant tissue. Hence, the need to further clarify the uptake of Cd with reliance to Zn-Si effects. Evidence from present study showed the overall Cd root to stem TF% and stem to leaf TF% was genotype dependent. Long-term exposure of Cd, Zn, and Si may result in a reduced root to stem Cd TF in the sensitive genotype [33]. Remarkably, at vegetative stage, the observed high Cd root uptake for Xiushui-110 than HIPJ-1 (Table 3) might explain the tolerance mechanism utilized by this genotype through attenuation of Cd root to stem translocation to localize much of the Cd in roots. The present study also confirmed this notion which show that rice plants hyper-accumulate Si for potential gain [46] From results, Si2 lowered Cd stem to leaf TF in Xiushui-110 than other treatments with Si1. We interpret the possibility of Cd localization in different plant parts as a response to Si augmentation subduing Cd movement. Si has been shown to lower Cd TF from root to shoot using nano-silicon forms with main possibilities being attributed to the co-precipitation of Cd with Si, thereby restricting the uptake and transport of Cd or the Cd localization in cell wall and vacuole, hence reducing Cd influx [8,18]. Evidence from present study also support vacuolar localization of Cd as a Cd root-shoot translocation deterrent by Si for treatment Cd1-Zn2-Si2 as compared to Cd1-Zn2-Si0 in HIPJ-1. This is in agreement with observed root ultrastructure for treatment Cd1-Zn2-Si2 with increased vacuolization (Figure 4). On the contrary, Zn significantly increased Cd TF in both genotypes. This could relate to a previous study on the possibility of Zn competing for phytochelatins (PC) with Cd leading to elevated levels of free Cd with rapid translocation [36,47], which could explain why Si mainly affected Cd translocation from stem to leaf in the present study.

Electron microscopy analysis assisted us to analyze the Cd, Zn, and Si effects at tissue level. Several studies have shown Cd to alter root cell division, thereby affecting plant growth [48,49]. The root apex produces new cells for root elongation and the present study results confirm the extremely altered root meristematic cells, cellular deformation and cell wall thinning because of Cd toxicity. Cd destroys the ultrastructure of nucleus and mitochondria by organelle swelling and disruption [8,50,51]. Similarly, in the present study, Xiushui-110 under alone Cd stress exhibited the absence of nucleus in the root tip cells, ultimately affecting cell division (Supplementary Figure S1), while the root tip cells of HIPJ-1 underwent destruction under the same treatment as compared to the corresponding control (Cd0-Zn0-Si0). The protective role of Si in ameliorating heavy metals such as Fe^2+^ toxicity includes root cell ultrastructure improvement by alleviating root apical cell destruction which also entailed vacuole formation and enlargement in observed ameliorated cells [52]. Likewise, the present study had increased Si improving cell formation with most cells acquiring a regular architecture and bearing a visible nucleolus, which was mainly observant in Xiushui-110 (Figure 4a). However, in HIPJ-1 the formation of large vacuoles was observed under Zn2 and Si2 combined treatment (Figure 4b). This was evident that Zn-Si combination was able to ameliorate Cd toxicity and improve root tip cell formation in HIPJ-1. Formation of large vacuoles in root tip cells for HIPJ-1 portrayed a distinct genotypic difference from Xiushui-110. The vacuolization could have alleviated Cd toxicity by sequestering the Cd inside the vacuoles, which could explain the Cd tolerance mechanism for this sensitive genotype. Vacuoles are termed as a major sink for heavy metal accumulation in plants subjected to metal stress [25,53]. The observed chloroplast ultrastructure closely correlated with *Pn* values. In the present study, a clear substantial damage can be observed in chloroplasts under Cd stress for both genotypes relative to control which is confirmed in previous study [42]. Plastoglobuli formation which also indicates plant stress was instigated by Cd as similarly observed in previous studies [40,48]. Nevertheless, an improvement in chloroplast formation was observed by the addition of Zn and Si, where different chloroplast architectures were produced for Xiushui-110 with an increased Si addition causing poor formation of granal thylakoids and fragmenting stromal thylakoids that became highly visible even though most of the chloroplasts maintained their rounded structure and plastoglobuli formation was reduced. The thinning of chloroplast membrane by increased Si, which literally caused the high visibility of granal thylakoids, could have greatly affected starch production in Xiushui-110. In HIPJ-1, high Zn (Zn2) and Si addition augmented starch grain formation in well-formed chloroplasts with few plastoglobuli, which could have been an influence that enabled positive growth.

## 4. Conclusions

The observed results clearly suggest the effectiveness of combined Zn-Si alleviation treatments in Cd stress reduction for both genotypes by increasing plant biomass due to an increased photosynthetic rate. The ultrastructural observations lead us to a conclusion that maintenance of chloroplast, increasing number of vacuoles and minimizing the mitochondrial disruption are the colossal factors behind Zn-Si ameliorative impact. Moreover, treatments of Zn and Si reduced root to the stem Cd translocation in HIPJ-1. The logical explanation behind this could be the mechanism utilized by this sensitive genotype, which employs Zn-Si combined association in order to restrict Cd root to stem transfer contrary to the tolerant genotype in a dose dependent manner. Zn and Si plausibly utilize other mechanisms to improve plant Cd resistance including reduction of lipid peroxidation as indicated by the reduction of MDA contents and increased antioxidant enzyme activities observed in this study. The findings of this study could assist further studies that may rely on sole and combined nutrient associations against Cd toxicity. This study will provide appropriate and valuable tool for elucidating the fundamental mechanisms responsible for heavy metal tolerance.

## 5. Material and Methods

### 5.1. Plant Material and Growth Conditions

Rice seeds of genotype Xiushui-110 (cadmium tolerant) and HIPJ-1 (cadmium sensitive), developed and verified by the Chinese National Rice Research Institute (CNRRI) Fuyang, Zhejiang, were grown in a controlled growth chamber at Zhejiang University, Zijingang Campus, Hangzhou, China. Seeds were first surface sterilized with 2% H_2_O_2_ for 15 min then washed with distilled water for 5 times and soaked for 2 days at 25 °C in darkness. Seeds were placed on germinating tray and covered with damp paper, then incubated at 30 °C in darkness for 24 h. Next, the germinating seeds were planted in sand and grown in a controlled growth chamber with 75–80% relative humidity, 30/22 °C (day/night) temperature, photoperiod of 16 h/8 h (light/dark) and light intensity of 225 ± 25 µmol m^−2^ s^−1^ for 14 d. Thereafter, seedlings were transplanted in 5 L plastic pot (5 seedlings per pot) and grown in nutrient solution (L^‒1^) which comprised of 2.9 mM NH_4_NO_3_; 1.7 mM MgSO_4_·7H_2_O; 1.0 mM K_2_SO_4_; 1.0 mM CaCl_2_; 0.32 mM NaH_2_PO_4_·2H_2_O; 36 μM EDTAFeNa; 18 μM H_3_BO_3_; 9.1 μM MnCl_2_·4H_2_O; 0.52 μM (NH_4_)_6_MoO_24_·4H_2_O; 0.16 μM CuSO_4_·5H_2_O; 0.15 μM ZnSO_4_·7H_2_O. The pH of nutrient solution was adjusted to 6.5–6.8 using HCl or NaOH as necessary [54]. The treatments comprised of 2 levels of Cd **{**Cd0 (0 μM); Cd1 (15 μM)**}** as CdCl_2_, 3 levels Zn **{**Zn0 (0 μM); Zn1 (1 μM); Zn2 (10 μM)**}** as ZnSO_4_·7H_2_O and 3 levels Si **{**Si0 (0 μM); Si1 (5 μM); Si2 (15 μM)**}** as Na_2_SiO_3_·9H_2_O. Zn, Cd and Si were administered into the nutrient solution in 7 treatment combinations along with control (Cd0-Zn0-Si0), i.e., Cd1-Zn0-Si0; Cd1-Zn2-Si0; Cd1-Zn0-Si2; Cd1-Zn1-Si1; Cd1-Zn2-Si1; Cd1-Zn1-Si2; Cd1-Zn2-Si2, and the plants were subjected to 30-d of treatment. Because EDTA readily forms complexes with Zn^2+^ thereby affecting Zn uptake, 36 μM FeCl_3_·6H_2_O was replaced with EDTAFeNa before applying treatments in nutrient solution. Experiment was triplicated following completely randomized block design. Nutrient solution was replaced twice a week.

### 5.2. Determination of Plant Morphological Attributes and Photosystem Parameters

Randomly selected seedlings were harvested at 30 days after treatment (DAT). Thereafter, plants were separated into roots and shoots; blot dried with paper towels, and fresh weight was measured immediately by electrical balance. Meanwhile, plant height was measured from plant base to top-most leaf tip. After drying in a hot air oven at 105 °C for 3 h and at 80 °C for 24 h [45] to a constant weight, roots and shoots were weighed again. Root length was measured by selecting the longest root from each plant’s base to the root tip. Photosynthestic rate (*Pn*), stomatal conductance (*Gs*), intercellular CO_2_ concentration, (*Ci*) and transpiration rate (*Tr*) were measured in vivo on the second leaf from apex using portable photosynthetic apparatus (Li-6400: LICOR, Lincoln, NE, United States of America). Relative chlorophyll contents (SPAD values) were measured using a chlorophyll meter (Minolta SPAD-502, Tokyo, Japan) from the second fully expanded leaves after 30 days of treatment according to [55].

### 5.3. Element Analysis

Approximately 0.1 g of dried root, stem-sheath, and shoot samples were placed in tubes to determine the concentrations of Cd, Zn, and Si. Then, 5 mL 65% HNO_3_ and 1 mL 30% *v*/*v* H_2_O_2_ were added into the dried samples and subsequently the samples were digested in a microwave (Mars 6, CEM Technologies, North Carolina, U.S.A). Thereafter, sample tubes were placed on a block heater for 2 h at 160 °C in a fume hood, afterwards diluted to 20 mL with Milli-Q water. The measurements took place at Zhejiang University, College of Life Sciences. The inductively coupled plasma–optical emission spectrometer (ICP-OES; Optima 8000DV; PerkinElmer) was used for root and shoot elements determination with the reference standard of 1000 mg L^−1^ of quality control standard of 21 elements supplied by PerkinElmer used for elemental contents detection [53]. The translocation factor (TF) of Cd from root to stem and stem to leaf was calculated using the following equation: Cd Root to Stem TF (%) = [Cd]_stem_/[Cd]_root_ × 100% and Cd Stem to Leaf TF (%) = [Cd]_leaf_/[Cd]_stem_ × 100% [33].

### 5.4. Determination of Antioxidant Enzyme Activity Assay

Fresh leaves and roots (0.5 g) were homogenized by grinding with pestle and mortar using 50 mM PBS (pH 7.0) in an ice bath. The mixture was centrifuged at 4 °C for 20 min at 12,000 rpm. Their supernatants were stored at −20 °C and used for the analyses of various antioxidant enzymes. The superoxide dismutase (SOD) and catalase (CAT) activities were determined according to [45,55]. SOD determination ensued after the supernatant was mixed in glass tubes with reaction solution prepared using 75 µM NBT, 20 µM riboflavin, 100 µM EDTA- Na_2_ and 130 mM methionine. Controls (light and dark) having reaction solution and distilled water were used for CK and zero reading respectively. The control light and all other samples were placed under light conditions at 4000 lux for 20 min while control dark sample in 100% dark condition, afterwards measured spectrophotometrically at 560 nm. Formula used is as follows: SOD activity (U g^‒1^ FW) = {(Ack-Ae) × V} ÷ {0.5 × Ack × W × Vt}. Where, Ae—OD value on the spectrophotometer; Ack—OD value for the control tube under light conditions (at 4000 lux for 20 min); V—total volume of the buffer solution used to extract the enzyme; W—fresh weight of the sample; and Vt—amount of enzyme extract used in reaction solution to test SOD.

CAT activity was measured by preparing a solution mix of 2.8 mL Tris HCL (1 M) buffer +0.1 mL of sample enzyme extract +0.1 mL of H_2_O_2_ (300 mM) was shaken gently and read spectrophotometrically at 240 nm within time range 0–30 s [56]. While the formula used for calculations is as follows: CAT (mM g^‒1^ FW) activity = (activity * A * V/a)/(E × W). Where, activity—OD value; W—fresh weight of the sample; V—total volume of the buffer solution used to extract the enzyme; a—amount of enzyme extract used in reaction solution to test; E—activity constant (39.4 mM cm^−1^).

### 5.5. Measurement of Malondialdehyde Contents

Malondialdehyde (MDA) concentration (nmol MDA g^−1^ FW) was measured and expressed as lipid peroxidation using a modified thiobarbituric acid (TBA) method according to previous studies [7,53]. Briefly, 0.5 g plant tissue sample was homogenized in a pre-cooled mortar on ice and 3 mL of 1 M Tris-HCL buffer (pH 7.4) was added and made up to 5 mL. After centrifugation of the samples at 12,000 rpm for 20 min at 4 °C, the enzyme extract (supernatant) was collected. MDA reaction solution comprised of 5% trichloro-acetic acid (TCA) and thiobarbituric acid (TBA). A solution mixture of 1.5 mL of sample enzyme extract +2.5 mL reaction solution was added in small tubes, placed in water bath at 95 °C for 15 min, and then immediately placed in an ice bath. The tubes were centrifuged at 4800 rpm for 10 min and read spectrophotometrically at 532 and 600 nm with distil water used for zero reading. MDA determination formula used; MDA (nmol g^−1^ FW) = [(OD532—OD600) × A × V] ÷ (a × E × W), where A—total RS + EE used; V—total volume of phosphate buffer (BPS) used for enzyme extraction; a—volume of the enzyme extract used; W—fresh weight of the sample and E—constant for MDA (1.55 × 10^−1^).

### 5.6. Ultrastructural Observations

Fresh root tips (meristematic cells) and leaf sections (1 mm^2^) from the mid of the third topmost leaf of 30-d treated rice plants from both genotypes were cut for TEM (transmission electron microscopy) studies [52]. Sample preparation started with double fixation. We fixed the samples with 2.5% glutaraldehyde in phosphate buffer (0.1 M, pH 7.0) for 6 h, and then washed three times in phosphate buffer (0.1 M, pH 7.0) for 15 min at each step. Sample post-fixation commenced with 1% OsO_4_ in phosphate buffer (0.1 M, pH 7.0) for 1–2 h and washing three times in phosphate buffer (0.1 M, pH 7.0) for 15 min at each step. Dehydration of samples utilized graded series of ethanol (30, 50, 70, 80, 85, 90, 95, and 100%) for 20 min at each step and transferring to absolute acetone for 20 min. After dehydration, the samples underwent infiltration. Thereafter, the samples were put in 1:1 mixture of absolute acetone and Spurr resin mixture for 1 h at room temperature, transferred to 1:3 mixture of absolute acetone and Spurr resin for 3 h and to final Spurr resin overnight. Afterwards, embedding and ultrathin sectioning produced the sample specimen. The specimen was transferred into an eppendorf containing Spurr resin and heated at 70 °C for more than 9 h. The specimen was then sectioned in LEICA EM UC7 ultratome and sections stained by uranyl acetate and alkaline lead citrate for 5–10 min, respectively. Sample specimen was observed in Hitachi Model H-7650 TEM.

### 5.7. Statistical Analysis

Experimental data were statistically analyzed using SAS university edition (3.5 basic ed., SAS Institute Inc., Cary, NC, USA), with the PROC ANOVA and PROC GLM procedures. Data obtained were evaluated using analysis of variance (ANOVA) at *p* < 0.05 significance levels. Mean values were compared using least significant difference (LSD) test at *p* < 0.05 [45,56]. Origin Pro version 2019b (Origin Lab Corporation, Wellesley Hills, Wellesley, MA, USA) was used to prepare graphs.

## Figures and Tables

**Table 1:** Effect of alone and combined cadmium, zinc, and silicon application (30 d) on plant growth parameters for two rice genotypes.

Genotype	Treatment	Root Length (cm)	Shoot Height (cm)	Root Dry Weight (g)	Stem-Sheath Dry Weight (g)	Shoot Dry Weight (g)
Xiushui-110	Cd0 Zn0 Si0	35.33 ± 1.52 ^a^	72.93 ± 1.67 ^a^	0.70 ± 0.04 ^a^	2.08 ± 0.09 ^a^	4.26 ± 0.27 ^a^
	Cd1 Zn0 Si0	25.33 ± 2.51 ^c^	43.33 ± 2.08 ^e^	0.37 ± 0.04 ^c^	0.58 ± 0.04 ^d^	1.09 ± 0.10 ^d^
	Cd1 Zn2 Si0	32.50 ± 3.00 ^ab^	55.50 ± 3.04 ^b^	0.69 ± 0.09 ^a^	1.08 ± 0.04 ^b^	3.18 ± 0.12 ^b^
	Cd1 Zn0 Si2	29.67 ± 2.08 ^b^	46.27 ± 0.25 ^de^	0.36 ± 0.06 ^c^	0.64 ± 0.01 ^cd^	1.21 ± 0.01 ^cd^
	Cd1 Zn1 Si1	29.70 ± 2.51 ^b^	47.17 ± 2.56 ^cde^	0.51 ± 0.12 ^b^	0.77 ± 0.10 ^c^	1.47 ± 0.15 ^c^
	Cd1 Zn2 Si1	25.83 ± 2.02 ^c^	51.00 ± 1.73 ^bcd^	0.38 ± 0.03 ^c^	0.74 ± 0.04 ^cd^	1.46 ± 0.05 ^c^
	Cd1 Zn1 Si2	30.70 ± 3.62 ^b^	51.83 ± 1.75 ^cb^	0.45 ± 0.01 ^bc^	0.71 ± 0.07 ^cd^	1.34 ± 0.07 ^cd^
	Cd1 Zn2 Si2	31.00 ± 1.73 ^b^	56.00 ± 1.81 ^b^	0.53 ± 0.10 ^b^	0.97 ± 0.19 ^b^	1.99 ± 0.14 ^b^
HIPJ-1	Cd0 Zn0 Si0	37.33 ± 1.15 ^a^	79.33 ± 2.25 ^a^	1.12 ± 0.07 ^a^	2.76 ± 0.20 ^a^	5.56 ± 0.45 ^a^
	Cd1 Zn0 Si0	13.67 ± 1.52 ^d^	41.50 ± 0.50 ^e^	0.30 ± 0.01 ^d^	0.44 ± 0.01 ^f^	0.82 ± 0.04 ^f^
	Cd1 Zn2 Si0	21.17 ± 1.49 ^bc^	56.83 ± 2.01 ^b^	0.44 ± 0.09 ^c^	0.83 ± 0.10 ^cd^	1.63 ± 0.23 ^cd^
	Cd1 Zn0 Si2	20.67 ± 1.23 ^c^	45.50 ± 0.50 ^d^	0.38 ± 0.11 ^cd^	0.56 ± 0.11 ^ef^	1.07 ± 0.26 ^ef^
	Cd1 Zn1 Si1	19.23 ± 0.68 ^c^	48.77 ± 0.75 ^cd^	0.50 ± 0.10 ^bc^	0.65 ± 0.05 ^def^	1.30 ± 0.13 ^def^
	Cd1 Zn2 Si1	20.16 ± 0.76 ^c^	52.00 ± 2.00 ^c^	0.45 ± 0.02 ^c^	0.94 ± 0.22 ^bc^	1.89 ± 0.25 ^bc^
	Cd1 Zn1 Si2	21.23 ± 1.32 ^bc^	47.50 ± 1.77 ^d^	0.51 ± 0.06 ^bc^	0.77 ± 0.04 ^cde^	1.5 ± 0.09 ^cde^
	Cd1 Zn2 Si2	24.33 ± 2.25 ^b^	57.17 ± 2.01 ^b^	0.59 ± 0.10 ^b^	1.09 ± 0.18 ^b^	3.18 ± 0.21 ^b^

Values with different letters in the same column per genotype are significantly different at *p* ≤ 0.05 probability level. Values (means, *n* = 3). Legends: Legends: Cd0 Zn0 Si0 (CK). Cd0 (0 μM), Cd1 (15 μM); Zn0 (0 μM), Zn1 (1 μM), Zn2 (10 μM); Si0 (0 μM), Si1 (5 μM), Si2 (15 μM).

**Table 2:** Effect of alone and combined cadmium, zinc and silicon application (30 d) on Zn, Cd, and Si uptake for two rice genotypes.

Genotype	Treatment	Zn (µg g^−1^ DW)			Cd (mg g^−1^ DW)			Si (mg g^−1^ DW)		
		Root	Stem-Sheath	Leaf	Root	Stem-Sheath	Leaf	Root	Stem-Sheath	Leaf
Xiushui-110	Cd0 Zn0 Si0	33.5 ± 0.01 ^d^	32.8 ± 0.01 ^b^	18.0 ± 0.01 ^e^	0 ^e^	0 ^f^	0 ^f^	0.11 ± 0.01 ^d^	0.28 ± 0.14 ^c^	0.20 ± 0.10 ^c^
	Cd1 Zn0 Si0	50.2 ± 0.02 ^cd^	15.5 ± 0.01 ^b^	19.9 ± 0.02 ^de^	1.54 ± 0.1 ^b^	0.13 ± 0.01 ^d^	0.07 ± 0.01 ^d^	0.15 ± 0.01 ^d^	0.59 ± 0.20 ^c^	0.43 ± 0.07 ^c^
	Cd1 Zn2 Si0	597.6 ± 0.05 ^b^	307.5 ± 0.18 ^a^	62.5 ± 0.04 ^b^	1.36 ± 0.02 ^c^	0.21 ± 0.02 ^c^	0.42 ± 0.01 ^a^	0.12 ± 0.01 ^d^	0.53 ± 0.09 ^c^	0.40 ± 0.04 ^c^
	Cd1 Zn0 Si2	60.0 ± 0.03 ^cd^	24.4 ± 0.01 ^b^	23.8 ± 0.01 ^d^	1.16 ± 0.06 ^d^	0.10 ± 0.01 ^e^	0.03 ± 0.01 ^e^	1.19 ± 0.26 ^bc^	4.809 ± 0.76 ^ab^	2.69 ± 0.18 ^b^
	Cd1 Zn1 Si1	121.4 ± 0.03 ^c^	52.3 ± 0.03 ^b^	32.5 ± 0.03 ^c^	1.34 ± 0.16 ^c^	0.14 ± 0.01 ^d^	0.15 ± 0.02 ^c^	1.21 ± 0.08 ^bc^	4.63 ± 0.61 ^ab^	2.81 ± 0.39 ^b^
	Cd1 Zn2 Si1	827.6 ± 0.11 ^a^	222.2 ± 0.05 ^a^	65.9 ± 0.02 ^ab^	1.77 ± 0.03 ^a^	0.27 ± 0.01 ^a^	0.35 ± 0.01 ^b^	0.96 ± 0.28 ^c^	4.47 ± 0.52 ^b^	3.69 ± 0.30 ^a^
	Cd1 Zn1 Si2	85.3 ± 0.01 ^cd^	42.9 ± 0.01 ^b^	29.8 ± 0.05 ^c^	1.19 ± 0.05 ^d^	0.10 ± 0.01 ^e^	0.09 ± 0.01^d^	1.52 ± 0.03 ^a^	5.42 ± 0.48 ^ab^	3.15 ± 0.17 ^ab^
	Cd1 Zn2 Si2	584.5 ± 0.03 ^b^	261.3 ± 0.03 ^a^	70.6 ± 0.15 ^a^	1.23 ± 0.01 ^cd^	0.24 ± 0.01 ^b^	0.06 ± 0.01 ^b^	1.32 ± 0.23 ^ab^	6.12 ± 0.50 ^a^	3.03 ± 0.62 ^ab^
HIPJ-1	Cd0 Zn0 Si0	29.4 ± 0.02 ^c^	87.1 ± 0.06 ^cd^	27.5 ± 0.01 ^b^	0 ^e^	0 ^e^	0 ^c^	0.15 ± 0.02 ^c^	0.45 ± 0.01 ^d^	0.25 ± 0.04 ^d^
	Cd1 Zn0 Si0	28.5 ± 0.02 ^c^	17.8 ± 0.01 ^e^	16.3 ± 0.01 ^cd^	1.04 ± 0.04 ^c^	0.11 ± 0.01 ^cd^	0.03 ± 0.01 ^b^	0.16 ± 0.04 ^c^	0.44 ± 0.20 ^d^	0.24 ± 0.02 ^d^
	Cd1 Zn2 Si0	378.7 ± 0.07 ^ab^	219.2 ± 0.05 ^a^	53.9 ± 0.03 ^a^	1.14 ± 0.01 ^abc^	0.25 ± 0.02 ^a^	0.35 ± 0.01 ^a^	0.02 ± 0.12 ^c^	0.54 ± 0.08 ^d^	0.19 ± 0.06 ^d^
	Cd1 Zn0 Si2	19.9 ± 0.01 ^c^	7.6 ± 0.01 ^e^	11.5 ± 0.02 ^d^	1.23 ± 0.16 ^ab^	0.89 ± 0.01 ^d^	0.05 ± 0.01 ^bc^	1.19 ± 0.02 ^a^	5.65 ± 1.03 ^ab^	3.26 ± 0.19 ^a^
	Cd1 Zn1 Si1	53.6 ± 0.11 ^c^	37.2 ± 0.02 ^de^	27.2 ± 0.02 ^bc^	1.03 ± 0.12 ^cd^	0.12 ± 0.01 ^c^	0.08 ± 0.02 ^b^	1.04 ± 0.04 ^ab^	3.09 ± 0.11 ^c^	1.48 ± 0.11 ^c^
	Cd1 Zn2 Si1	453.1 ± 0.12 ^a^	141.1 ± 0.01 ^bc^	49.6 ± 0.09 ^a^	1.23 ± 0.19 ^a^	0.27 ± 0.02 ^a^	0.30 ± 0.01 ^a^	0.84 ± 0.19 ^b^	4.30 ± 0.63 ^bc^	1.75 ± 0.16 ^b^
	Cd1 Zn1 Si2	67.2 ± 0.03 ^c^	32.4 ± 0.01 ^de^	21.1 ± 0.01 ^bcd^	0.86 ± 0.10 ^d^	0.10 ± 0.01 ^cd^	0.07 ± 0.01 ^b^	1.00 ± 0.01 ^ab^	2.98 ± 0.04 ^c^	1.69 ± 0.10 ^bc^
	Cd1 Zn2 Si2	364.5 ± 0.06 ^b^	171.6 ± 0.02 ^ab^	44.6 ± 0.01 ^a^	1.05 ± 0.01 ^bc^	0.19 ± 0.04 ^b^	0.28 ± 0.02 ^a^	1.01 ± 0.03 ^ab^	5.86 ± 1.54 ^a^	3.23 ± 0.27 ^a^

Values with different letters in the same column per genotype are significantly different at *p* ≤ 0.05 probability level. Values (means, *n* = 3). Legends: Legends: Cd0 Zn0 Si0 (CK). Cd0 (0 μM), Cd1 (15 μM); Zn0 (0 μM), Zn1 (1 μM), Zn2 (10 μM); Si0 (0 μM), Si1 (5 μM), Si2 (15 μM).

**Table 3:** Effect of alone and combined cadmium, zinc, and silicon application (30 d) on cadmium translocation factor for two rice genotypes.

Treatment	Cd Root to Stem TF, %		Cd Stem to Leaf TF, %	
	Xiushui-110	HIPJ-1	Xiushui-110	HIPJ-1
Cd0 Zn0 Si0	0 ^e^	0 ^e^	0 ^g^	0 ^e^
Cd1 Zn0 Si0	8.25 ± 0.71 ^d^	10.14 ± 0.81 ^cd^	5.55 ± 0.547 ^e^	2.62 ± 0.38 ^de^
Cd1 Zn2 Si0	15.24 ± 1.60 ^b^	21.96 ± 1.98 ^a^	20.48 ± 2.21 ^a^	13.74 ± 2.79 ^a^
Cd1 Zn0 Si2	8.86 ± 0.15 ^d^	7.17 ± 0.86 ^d^	2.51 ± 1.58 ^f^	1.52 ± 1.91 ^e^
Cd1 Zn1 Si1	10.34 ± 0.43 ^c^	13.10 ± 1.72 ^c^	10.46 ± 1.08 ^cd^	6.26 ± 1.93 ^bc^
Cd1 Zn2 Si1	15.63 ± 0.57 ^b^	23.25 ± 2.52 ^a^	13.46 ± 1.51 ^c^	9.42 ± 1.96 ^b^
Cd1 Zn1 Si2	8.56 ± 0.49 ^d^	13.22 ± 1.57 ^c^	8.39 ± 0.53 ^d^	5.19 ± 1.88 ^cd^
Cd1 Zn2 Si2	19.33 ± 1.08 ^a^	18.16 ± 0.10 ^b^	15.96 ± 1.05 ^b^	14.46 ± 2.93 ^a^

Values with different letters in the same column per genotype are significantly different at *p* ≤ 0.05 probability level. Values (means, *n* = 3). Legends: Legends: Cd0 Zn0 Si0 (CK). Cd0 (0 μM), Cd1 (15 μM); Zn0 (0 μM), Zn1 (1 μM), Zn2 (10 μM); Si0 (0 μM), Si1 (5 μM), Si2 (15 μM).

## Data Availability

The data presented in this study are available on request from the corresponding author.

## Supplementary Materials

The following are available online at https://www.mdpi.com/2223-7747/10/1/87/s1, Supplementary Table S1. Effect of alone and combined cadmium, zinc, and silicon application (30 d) on tillering and leaf dry weight for two rice genotypes. Supplementary Table S2. Effect of alone, and combined cadmium, zinc, and silicon application (30 d) on root, stem-sheath and leaf Ca, Mg, K, and Fe mineral elemental concentration for two rice geno-types. Supplementary Figure S1. Ultrastructure micrograph images (a) Xiushui-110 (b) HIPJ-1after 30-d treatments. La-bels A and B (meristematic cells at root tip), C and D (leaf chloroplast). Cd0 (0 μM), Cd1 (15 μM); Zn0 (0 μM), Zn1 (1 μM), Zn2 (10 μM); Si0 (0 μM), Si1 (5 μM), Si2 (15 μM). CH- chloroplast, STH- stromal thylakoids, GTH-granal thylakoids, SG- starch granule, P-plastoglobuli, CW-cell wall, N-nucleus, NU- Nucleolus, VA-vacuole.
