# Supplementary material for: Modulation of Key Physio-Biochemical and Ultrastructural Attributes after Synergistic Application of Zinc and Silicon on Rice under Cadmium Stress
Source: Plants (Basel). 2021 Jan 4;10(1):87. doi: 10.3390/plants10010087 (PMC7824249; doi:10.3390/plants10010087)
Supplement: Supplementary file 1 [file plants-10-00087-s001.pdf]

Table S1. Effect of alone and combined cadmium, zinc and silicon application (30 d) on tillering and leaf dry weight for two rice genotypes.

Genotype	Treatment	Tillers (average $plant^{-1}$)	Leaf Dry weight (g)
**Xiushui-110**	Cd0 Zn0 Si0	4.08±0.52 a	3.18±0.19 a
	Cd1 Zn0 Si0	1.75±0.25 d	0.52±0.06 d
	Cd1 Zn2 Si0	3.67±0.28 a	1.10±0.09 b
	Cd1 Zn0 Si2	2.00±0.10 cd	0.56±0.05 cd
	Cd1 Zn1 Si1	3.33±0.14 bc	0.70±0.6 cd
	Cd1 Zn2 Si1	3.25±0.43 bcd	0.73±0.02 c
	Cd1 Zn1 Si2	2.00±0.42 cd	0.63±0.01 cd
	Cd1 Zn2 Si2	2.75±0.25 b	1.02±0.12 b
**HIPJ-1**	Cd0 Zn0 Si0	4.75±0.42 a	2.81±0.25 a
	Cd1 Zn0 Si0	1.69±0.33 d	0.38±0.04 e
	Cd1 Zn2 Si0	2.83±0.52 bc	0.81±0.12 bcd
	Cd1 Zn0 Si2	3.33±0.57 cd	0.51±0.14 de
	Cd1 Zn1 Si1	2.56±0.19 c	0.71±0.13 cd
	Cd1 Zn2 Si1	3.50±0.17 b	0.95±0.23 bc
	Cd1 Zn1 Si2	3.44±0.50 cd	0.78±0.04 cd
	Cd1 Zn2 Si2	3.33±0.57 b	1.10±0.20 b

Values with different letters in the same column per genotype are significantly different at $P \leq 0.05$ probability level. Values (means, n=3). Legends: Legends: Cd0 Zn0 Si0 (CK). Cd0 (0 µM), Cd1 (15 µM); Zn0 (0 µM), Zn1 (1 µM), Zn2 (10 µM); Si0 (0 µM), Si1 (5 µM), Si2 (15 µM).

Table S2. Effect of alone and combined Cd, Zn and Si application (30 d) on root, stem sheath and leaf mineral elemental concentration of two rice genotypes.

Genotype	Treatment	Ca (mg g^{-1} DW)			Mg (mg g^{-1} DW)			K (mg g^{-1} DW)			Fe (mg g^{-1} DW)		
		Root	Stem-Sheath	Leaf	Root	Stem-Sheath	Leaf	Root	Stem-Sheath	Leaf	Root	Stem-Sheath	Leaf
**Xiushui-110**	Cd0 Zn0 Si0	2.78±0.15 cd	1.54±0.09 d	3.60±0.21 abc	1.69±0.21 c	3.34±0.19 d	4.22±0.41 bc	17.93±3.86 c	49.71±3.22 a	30.30±2.75 ab	2.91±1.76 cd	0.29±0.04 a	0.23±0.01 a
	Cd1 Zn0 Si0	4.37±0.21 b	2.96±0.29 b	3.82±0.07 ab	1.95±0.20 bc	4.48±0.45 cd	3.76±0.31 c	19.98±2.23 bc	37.54±4.43 c	30.38±3.09 ab	4.74±0.44 abc	0.06±0.01 c	0.73±0.01 cd
	Cd1 Zn2 Si0	2.35±0.05 d	1.79±0.10d	3.18±0.56 c	2.63±0.27 a	6.70±1.17 a	4.32±0.30 abc	25.33±1.03 a	53.65±0.49 a	32.12±3.24 a	4.70±1.06 abc	0.10±0.01 b	0.75±0.01 cd
	Cd1 Zn0 Si2	6.02±1.11 a	3.69±0.79 a	3.65±0.11 abc	2.03±0.07 bc	6.53±0.85 ab	4.83±0.42 a	20.12±0.60 bc	37.18±3.68 c	32.65±1.95 a	5.24±0.68 ab	0.61±0.01 c	0.52±0.01 e
	Cd1 Zn1 Si1	3.22±0.06 cd	2.91±0.62 b	3.65±0.39 abc	2.17±0.54 abc	5.35±0.61ab c	4.70±0.26 ab	19.53±2.53 bc	46.99±5.82 ab	32.98±2.57 a	4.06±0.19 bcd	0.07±0.01 bc	0.50±0.01 e
	Cd1 Zn2 Si1	2.62±1.21 cd	1.58±0.08 d	3.98±0.32 a	2.14±0.19 abc	5.08±1.71 bc	4.71±0.15 ab	22.34±3.11 ab	49.71±2.76 a	31.94±1.24 ab	5.65±0.73 a	0.09±0.01 bc	0.79±0.02 c
	Cd1 Zn1 Si2	3.53±0.02 bc	2.18 ±0.36 cd	3.36±0.11 bc	2.39±0.39 ab	5.57±0.94 abc	4.39±0.13 ab	19.39±1.98 bc	40.72±4.12 bc	27.19±4.49 ab	5.10±0.36 abc	0.77±0.02 bc	0.65±0.01 d
	Cd1 Zn2 Si2	2.86±0.05 cd	2.83±0.14 bc	3.26±0.58 bc	2.10±0.31 abc	5.90±.032 abc	3.78±0.48 c	21.65±1.95 abc	51.46±4.17 a	33.18±2.11 a	3.67±0.46 c	0.10±0.01 b	0.93±0.01 b
**HIPJ-1**	Cd0 Zn0 Si0	1.64±0.02 b	1.41±0.04 d	3.87±0.22 a	1.43±0.22 c	3.66±0.42 d	4.84±0.17 a	14.62±0.50 d	54.11±4.67 a	23.37±0.12 e	3.06±0.40 a	0.33±0.04 a	0.23±0.01 a
	Cd1 Zn0 Si0	4.67±1.27 a	2.91±0.34 b	3.55±0.43 ab	2.42±0.23 ab	5.42±1.23 bc	4.09±0.55 cd	18.99±0.63 b	37.89±2.84 c	27.38±1.07 d	1.97±0.02 bc	0.64±0.01 b	0.43±0.01 c
	Cd1 Zn2 Si0	1.92±0.21 b	1.79± 0.30 cd	3.05±0.27 bc	2.88±0.46 a	4.74±0.59 bcd	3.58±0.04 de	24.24±1.51 a	51.82±2.03 a	29.95±0.71 b	1.68±0.28 cd	0.06±0.01 b	0.75±0.01 b
	Cd1 Zn0 Si2	2.71±0.70 b	4.33±0.19a	3.69±0.71 ab	2.56±0.18 ab	6.99±0.04 a	4.72±0.45 ab	17.67±1.50 bc	41.90±0.78 c	32.74±0.22 a	3.14±0.21 a	0.60±0.03 b	0.50±0.01 c
	Cd1 Zn1 Si1	2.30±0.71 b	2.43±0.41 bcd	2.83±0.18 c	2.64±0.34 a	5.78±1.40 ab	3.39±0.14 e	19.53±1.30 b	46.74±1.36 b	29.87±3.21 bc	1.91±0.02 c	0.58±0.01 b	0.58±0.02 c
	Cd1 Zn2 Si1	2.33±0.06 b	2.49±1.51 bc	2.79±0.45 c	2.48±0.29 ab	3.98±0.02 cd	3.61±0.28 de	19.65±1.95 b	55.35±1.94 a	27.62±0.13 cd	1.65±0.28 cd	0.49±0.00 b	0.78±0.02 b
	Cd1 Zn1 Si2	2.82±0.57 b	1.88±0.28 cd	3.31±0.21 abc	2.14±0.01 b	4.70±1.31 bcd	4.31±0.01 bc	18.90±1.48 b	55.16±1.33 a	29.70±1.34 bc	2.38±0.26 b	0.50±0.01 b	0.52±0.01 c
	Cd1 Zn2 Si2	5.33± 1.07a	2.12±0.05 bcd	2.86±0.18 c	2.75±0.33 a	4.32±0.45 bcd	3.47±0.23 e	16.42±0.61 cd	55.32±1.90 a	31.74±0.34 ab	1.38±0.27 d	0.72±0.02 b	0.82±0.01 b

Values with different letters in the same column per genotype are significantly different at $P \leq 0.05$ probability level. Values (means, n=3). Legends: Legends: Cd0 Zn0 Si0 (CK). Cd0 (0 µM), Cd1 (15 µM); Zn0 (0 µM), Zn1 (1 µM), Zn2 (10 µM); Si0 (0 µM), Si1 (5 µM), Si2 (15 µM). DW; Dry weight.
